# Parametric Finite Element Evaluation of Load Redistribution Under Progressive Lumbar Disc Degeneration

**DOI:** 10.3390/bioengineering13020234

**Published:** 2026-02-17

**Authors:** Oleg Ardatov, Sofia Rita Fernandes, Artūras Kilikevičius, Vidmantas Alekna

**Affiliations:** 1 Faculty of Medicine, Vilnius University, LT-03101 Vilnius, Lithuania; vidmantas.alekna@mf.vu.lt; 2 Faculty of Mechanics, Vilnius Gediminas Technical University, LT-10223 Vilnius, Lithuania; arturas.kilikevicius@vilniustech.lt; 3 Instituto de Biofísica e Engenharia Biomédica, Faculdade de Ciências, Universidade de Lisboa, 1749-016 Lisboa, Portugal; srcfernandes@ciencias.ulisboa.pt

**Keywords:** annulus fibrosus, degenerative spinal diseases, finite element method, intervertebral discs, material modeling, nucleus pulposus, numerical modeling, spinal biomechanics

## Abstract

This study presents a finite element (FE) investigation of intervertebral disc (IVD) degeneration in the human lumbar spine (L1–L3 segment). The model, based on CT-derived geometry and isotropic hyperelastic representation of disc tissues, incorporates controlled simplifications, detailed in the limitations section. Degenerative changes were parametrically simulated across healthy, mild, moderate, and severe stages by reducing disc height (up to 60%), nucleus pulposus volume (up to 70%), and adjusting tissue stiffness to reflect dehydration and fibrosis. Displacement-controlled compressive loading was applied to assess von Mises stress distributions, reaction forces, and load transfer mechanisms. Results indicate significant load redistribution: annulus fibrosus stresses increased by up to 175% in severe degeneration, while nucleus pulposus stresses decreased by ~70%, indicating a diminished compressive load-bearing contribution of the nucleus. Model predictions were validated against cadaveric and in vivo data, confirming trends in intradiscal pressure (IDP) reductions (40–70%) and stress elevations. The parametric framework elucidates interactions between geometric and material changes, providing clinicians with insights into degeneration progression and guiding biomedical engineers in implant design and interventions.

## 1. Introduction

Degeneration of the intervertebral disc involves structural and biochemical alterations that impair its mechanical function. Key manifestations include reduced water and proteoglycan content, loss of disc height, annular tears, endplate sclerosis, and osteophyte formation, all of which influence spinal load distribution and mobility [1].

One of the earliest degenerative changes is the decreased hydration of the nucleus pulposus due to a decline in proteoglycan concentration. Water content, which is approximately 85% in young individuals, drops to about 75% in older age, accompanied by a reduction in osmotic pressure from ~0.19 MPa at age 37 to ~0.05 MPa at age 91 [2]. This diminishes the disc’s ability to absorb loads and maintain uniform pressure distribution.

Progressive matrix degradation also alters tissue mechanical properties. Early degeneration may increase segmental flexibility due to reduced intradiscal pressure and annular weakening. At advanced stages, fibrosis and calcification lead to tissue stiffening and restricted mobility [2,3]. Accordingly, the elastic modulus decreases in the nucleus pulposus, while the annulus fibrosus may exhibit either reduced elasticity or pathological stiffening depending on the degeneration stage.

Geometric changes in the disc include a reduction in its height, which is particularly pronounced in the later stages of degeneration. Normally, even with aging, the height of the intervertebral disc remains relatively stable or may even slightly increase, but in pathological degeneration, a significant reduction in disc space height occurs, leading to decreased mobility of the spinal segment and alterations in the mechanics of adjacent vertebrae. The loss of height is associated with both dehydration of the nucleus pulposus and the destruction of structural elements in the annulus fibrosus. Additionally, thinning and weakening of the annulus fibrosus occur, increasing the risk of tears and fissures [4]. The studies [5,6,7] also provide specific data on the progressive reduction in intervertebral disc height due to degeneration. In cases of mild degeneration, the disc height decreases by 20%, while in moderate degeneration, the reduction reaches 40%. In severe degeneration, the height loss becomes even more pronounced, reaching 60%. These structural changes are accompanied by a reduction in the volume of the nucleus pulposus, leading to an increased load on the annulus fibrosus and alterations in the distribution of mechanical forces within the spinal segment.

Numerical modeling, particularly the finite element method, has proven to be an essential tool in studying the biomechanics of intervertebral discs, providing detailed insights into mechanical responses under various loading scenarios, tissue configurations, and degenerative conditions challenging to replicate experimentally [8,9]. Zhu et al. [10] utilized FE modeling to explore how disc degeneration affects the mechanical and electrical signals at the interface between the disc and vertebral bodies, highlighting its importance for understanding vertebral adaptation and remodeling processes. Holzapfel and Ogden [11] introduced an analytical model accounting for residual stresses and their effect on anisotropic disc behavior under different loading scenarios. Ghezelbash et al. [12] and Kandil et al. [13] adopted advanced microstructural-based modeling approaches to accurately represent the heterogeneous collagen and elastic fiber networks of the annulus fibrosus, demonstrating the necessity of detailed structural modeling to accurately reproduce mechanical disc responses. Xi et al. [14] examined the effects of fibrosis and disc-height reduction, concluding that these factors collectively alter load distributions, promoting vertebral osteoporosis and joint degeneration.

Despite extensive FE research on lumbar biomechanics, many degeneration models vary only one factor at a time (e.g., disc height, nucleus dehydration, or tissue stiffness) rather than combining these changes within a unified parametric framework. This limits assessment of how degenerative mechanisms interact to drive load redistribution.

To address this gap, we propose an integrated parametric degeneration model that simultaneously varies disc height (20–60%), nucleus pulposus volume (25–70%), and soft-tissue stiffness across four stages. The main contribution is a sensitivity-aware, mechanically consistent framework for comparative trends rather than identification of new biomechanical phenomena.

Applied to a two-level L1–L3 segment with L2–L3 kept intact as an internal reference, the approach enables systematic quantification of coupled geometric–material effects, including adjacent-segment interactions. Verification against in vivo and cadaveric trends, together with sensitivity analysis, supports robustness. Overall, the framework is reproducible and modular for comparative biomechanical analysis and engineering applications such as implant design.

## 2. Materials and Methods

### 2.1. Initial Data and Numerical Model Development

The numerical model was developed using computed tomography scans of a sixty-year-old woman exhibiting typical age-related degenerative changes and involved several sequential steps. Initially, using the 3D Slicer program [15], the projections were aligned into three-dimensional geometry of the vertebral bodies (L1–L3), which was then converted to STL format. CT data were processed using threshold-based segmentation to isolate bony structures (global threshold range 200–2000 HU), followed by manual refinement around the endplates and pedicles. During the segmentation of the bony components, special attention was given to approximate the patient-specific sagittal curvature of the L1–L3 segment based on the midsagittal CT contours, while mesh smoothing was kept minimal to avoid altering the overall alignment. After extracting the L1–L3 vertebrae, surface meshes were decimated to reduce the initial number of elements (from approximately 60,000 to 39,000 per vertebra) while preserving anatomical fidelity. Subsequently, using the MeshLab 2022.22 software package [16], the geometry underwent noise reduction and smoothing. Mesh smoothing was performed using a Laplacian smoothing filter (2 iterations, relaxation factor 0.3) to remove noise artefacts without altering the overall geometry. Then, the processed STL file was exported to the SolidWorks 2024 software package [17], where using the 3D Scan module, the external geometry of the vertebrae was processed further. In SolidWorks, the model was refined by creating an internal continuous cavity representing cancellous bone, while assigning a thickness of 0.5 mm to the outer shell reflecting cortical bone [18]. The intervertebral discs (L1–L2 and L2–L3), consisting of the annulus fibrosus and the nucleus pulposus, were subsequently added using SolidWorks Features tool. The heights of the intervertebral discs were set to 10 mm, which corresponds to the average height of a healthy intervertebral disc [19]. At this stage of modeling, special attention was paid to accurately represent the cartilaginous endplates, which were assigned a uniform thickness of 0.5 mm [18]. The intact endplate assumption was adopted to isolate the mechanical effects of disc degeneration from endplate-related pathology. While endplate sclerosis commonly accompanies advanced degeneration and would locally increase stiffness and alter stress transfer to the trabecular bone, modeling it would introduce additional parametric uncertainty (e.g., sclerosis extent, mineralization degree). By keeping the endplate unchanged across all stages, we ensured that observed stress redistribution patterns are attributable solely to disc geometric and material changes. This simplification may underestimate local stress concentrations at the bone–disc interface in moderate and severe cases but does not affect the comparative trends central to this study.

**Figure 1:**
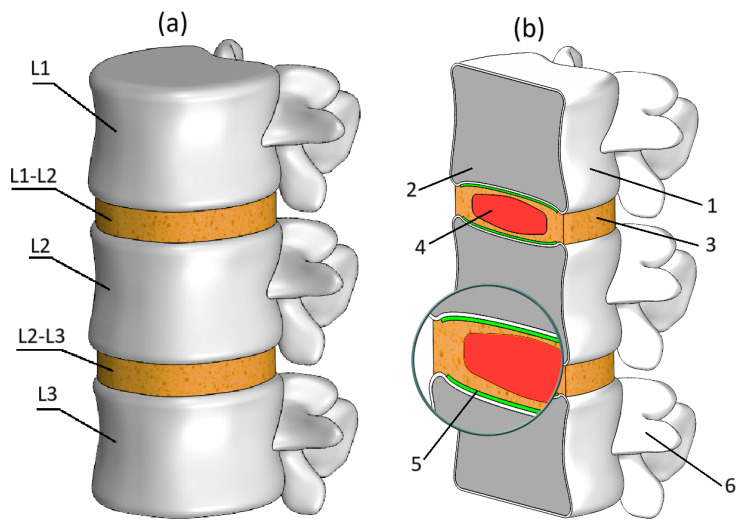
(**a**) Trimetric view of numerical lumbar spine model; (**b**) Section view of the model: 1—cortical shell, 2—cancellous bone, 3—annulus fibrosus, 4—nucleus pulposus, 5—bony endplate, 6—posterior elements.

Another notable simplification concerns the trabecular tissue representation. In this model, trabecular bone is depicted as a continuous solid body, visible in cross-section (Figure 1b). This discrepancy from actual anatomy is partially offset by reducing the assigned modulus of elasticity, as detailed further in Section 2.3. This approach captures the macroscopic load-bearing contribution of trabecular bone while avoiding the complexity and uncertainty associated with patient-specific trabecular microarchitecture. As the study focuses on comparative behavior between degeneration stages, this level of approximation is sufficient for the intended analysis. An additional significant simplification pertains to the perfectly flat surfaces at the external boundaries, specifically the lower endplate of vertebra L3 and the upper surface of vertebra L1, clearly illustrated in Figure 1b. This simplification was deliberately chosen to facilitate stable fixation and effective application of compressive loading, which constitutes the focus of this study. Conversely, the vertebral endplate surfaces adjacent to the intervertebral discs L1–L2 and L2–L3 were modeled with particular care, preserving their natural curvature due to the substantial impact this geometry has on biomechanical behavior.

### 2.2. Modelling the Geometrical Impact of Degenerative Changes on IVD

In the present study, the geometrical effects of intervertebral disc degeneration were modeled at the L1–L2 level by modifying two primary morphological parameters: disc height and nucleus pulposus volume. Specifically, mild, moderate, and severe degenerative stages correspond to reductions in disc height by 20%, 40%, and 60%, respectively, compared to a healthy intervertebral disc [4]. Both anterior and posterior disc heights were decreased proportionally during the modelling process. The reduction in disc height was applied uniformly across the anterior–posterior direction to represent the predominant pattern of symmetric disc space narrowing commonly observed in early-to-moderate degenerative disc disease without significant sagittal imbalance or endplate deformity. While advanced degeneration may sometimes produce anterior wedge-shaped collapse (particularly in cases with kyphotic deformity or asymmetric endplate damage), uniform reduction was chosen here to maintain comparability across degeneration stages and to focus on the direct biomechanical consequences of height and volume loss rather than postural adaptations.

The percentage reductions in disc height and nucleus pulposus volume were selected within the empirical ranges reported in radiological and MRI-based studies of lumbar degeneration, where advanced stages commonly exhibit marked height loss and nucleus shrinkage [5,6,7]. These values were therefore selected to reflect typical structural progression rather than patient-specific anatomy. The use of four sequential degeneration stages also provides an inherent sensitivity-type assessment of how progressively increasing geometric and material alterations affect mechanical response.

The L2–L3 disc was intentionally kept unchanged and served as an internal reference, allowing us to clearly identify the mechanical effects produced solely by the degenerative alterations at L1–L2.

**Figure 2:**
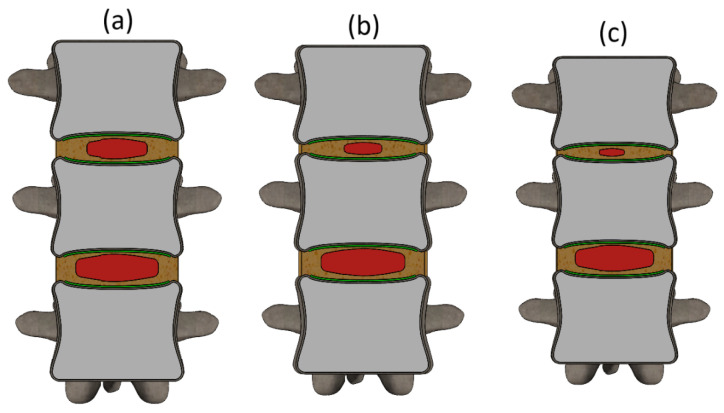
Different stages of the L1–L2 IVD degeneration (sectioned frontal view): (**a**) Mild degeneration; (**b**) Moderate degeneration; (**c**) Severe degeneration.

Furthermore, with the progression of degeneration, the cross-sectional area (on the horizontal plane) of nucleus pulposus is reduced by 25% (mild degeneration), 50% (moderate degeneration), and ultimately to 70% (severe degeneration). The cross-sectional views of the degenerative models are presented in Figure 2.

As seen in Figure 2, each stage of degeneration is characterized by progressive reductions in disc height and nucleus pulposus volume. In model 2a (mild degeneration), the disc height is slightly reduced, and the nucleus pulposus retains most of its volume, occupying a substantial portion of the disc space. In model 2b (moderate degeneration), the reduction in disc height becomes more pronounced, and the nucleus pulposus is visibly smaller in cross-sectional area. Finally, in model 2c (severe degeneration), there is a substantial collapse of the intervertebral disc space, and the nucleus pulposus is significantly diminished, appearing as a narrow horizontal structure.

Anterior and posterior height reductions were applied proportionally as a modeling simplification consistent with the percentage-based parametrization of disc height loss. This approach ensured controlled geometric modification without introducing additional asymmetry-related effects.

### 2.3. Mechanical Properties of Model Components

Bone structures as well as the cartilaginous endplates were modeled as perfectly elastic, homogeneous continuum materials. The mechanical properties assigned to these components, such as Young’s modulus and Poisson’s ratio, were selected based on consistently reported values in previously published studies [4,18,20] and are summarized in Table 1.

The soft tissues (annulus fibrosus and nucleus pulposus) were modelled as hyperelastic isotropic bodies. The Mooney–Rivlin (M-R) material model was applied, where M-R strain energy density function is defined as a two-constant formulation [21]:

(1) $$w=C_{1}\left(I_{1}-3\right)+C_{2}\left(I_{2}-3\right)+\frac{1}{2}K{\left(I_{3}-1\right)}^{2},$$

where *C*_1_ and *C*_2_ are the first and second material constants, respectively, associated with the distortion response, while *K* is the material constant governing the volumetric response. The terms *I*_1_, *I*_2_ and *I*_3_ represent the reduced invariants of the Cauchy–Green deformation tensor, defined through the principal stretch ratios.

The volumetric material constant *K* can be expressed as follows:

(2) $$K=\frac{6(C_{1}+C_{2})}{3(1-2\upsilon )},$$

where υ represents the Poisson’s ratio.

The annulus fibrosus was modeled as an isotropic hyperelastic material (Mooney–Rivlin), which is a major simplification compared to its real anisotropic fiber-reinforced structure. Comparative studies indicate that isotropic or simplified embedded-fiber annulus formulations can be less accurate than fully anisotropic models for predicting absolute range of motion (R^2^ = 0.69–0.89 vs. 0.95) and intradiscal pressure (R^2^ = 0.80–0.86 vs. 0.87) [12,22]. However, the largest reported discrepancies in local stress–strain fields (up to 16-fold) are largely associated with differences in specimen geometry and boundary conditions, rather than isotropy alone [12,22]. Accordingly, when the same isotropic constitutive model and loading setup are applied consistently across all degeneration stages—as in the present study—this simplification is expected to preserve comparative degeneration-driven trends, while absolute peak stresses remain sensitive to constitutive choices; for example, peak AF von Mises stresses may vary by >100% between hyperelastic formulations [23].

The degree of degeneration of the annulus fibrosus and nucleus pulposus was simulated by altering the elastic constants, assuming that higher effective stiffness corresponds to a more deteriorated tissue state [24,25]. The range of elastic constants for cartilage was derived from previously reported studies [19,26,27,28,29]. The values of the constants *C*_1_ and *C*_2_ are summarized in Table 2. The Poisson’s ratio was considered constant and set at 0.45 for the annulus fibrosus [30] and 0.4995 for the nucleus pulposus [30].

It should be noted that material properties for model components were taken from experimentally established studies commonly used in FE modeling of the lumbar spine. Because the literature lacks a unified, age- and sex-specific dataset, values were selected from sources reporting physiologically consistent ranges rather than matched patient-specific properties.

### 2.4. Problem Formulation

To accurately capture the mechanical response of the lumbar spine—especially under conditions involving large deformations and the nonlinear material behavior characteristic of intervertebral disc tissues—the framework of nonlinear elasticity was employed in this study. This approach enables a more realistic simulation of the complex stress–strain relationships occurring within hyperelastic soft tissues, such as the annulus fibrosus and nucleus pulposus, when subjected to physiological loading.

In this study a nonlinear static analysis was performed. Although the solver internally uses the standard dynamic equilibrium formulation (including mass and damping matrices), inertial and damping terms were suppressed, and the solution proceeded through incremental load steps combined with the Newton–Raphson iterative method:

(3) [K](i)t+∆t∆U(i)={R}(i)t+∆t,

where *^t^*^+∆*t*^{*R*}^(*i*)^ denotes the effective load vector, [∆*U*]^(*i*)^ is the vector of incremental displacements at iteration (*i*) and *^t^*^+∆*t*^[*K*]^(*i*)^ represents the effective stiffness matrix. The three-dimensional nonlinear analysis was performed using Intel Direct Sparse solver.

It should be noted that the time increments reported by the software [17] correspond to load-increment steps, not to physical time integration. At each increment, the stiffness matrix was updated, and the equilibrium was solved in a fully nonlinear manner.

To assess the stress distribution within the intervertebral disc and surrounding bony structures, the von Mises stress criterion was employed. Although originally developed for isotropic, ductile metals, this scalar metric has gained widespread acceptance in biomechanical simulations [18,19,20,31,32,33,34,35,36] due to several advantages. First, von Mises stress provides a single-value representation of the multi-axial stress state, facilitating comparative analysis across complex anatomical regions. Second, despite the anisotropic and heterogeneous nature of biological tissues, studies [31,35,36] have demonstrated that von Mises stress correlates well with regions prone to mechanical failure or tissue remodeling, particularly in cortical and trabecular bone, as well as in the annulus fibrosus under compression and bending.

In the context of intervertebral discs, where large deformation gradients occur, von Mises stress remains a practical indicator for identifying peak stress zones and assessing the structural integrity of hyperelastic materials [31,32,33,34]. It is also widely used for evaluating load-bearing performance in bone tissue, highlighting regions susceptible to micro-damage accumulation or structural failure [34]. At the same time, von Mises stress does not capture direction-dependent fiber mechanics of the annulus fibrosus and therefore cannot fully describe anisotropic tissue behavior. For this reason, it is interpreted here as a relative indicator rather than a physiologically complete measure.

The von Mises criterion is expressed in Equation (4):

(4) $$\sigma_{y}=\sqrt{\frac{{\left(\sigma_{1}-\sigma_{2}\right)}^{2}+{\left(\sigma_{2}-\sigma_{3}\right)}^{2}+{\left(\sigma_{3}-\sigma_{1}\right)}^{2}}{2}},$$

where *σ*_1_, *σ*_2_ and *σ*_3_ are the maximum, intermediate, and minimum principal stresses respectively, and *σ_y_* is a von Mises stress.

### 2.5. Boundary Conditions and Finite Element Mesh

**Figure 3:**
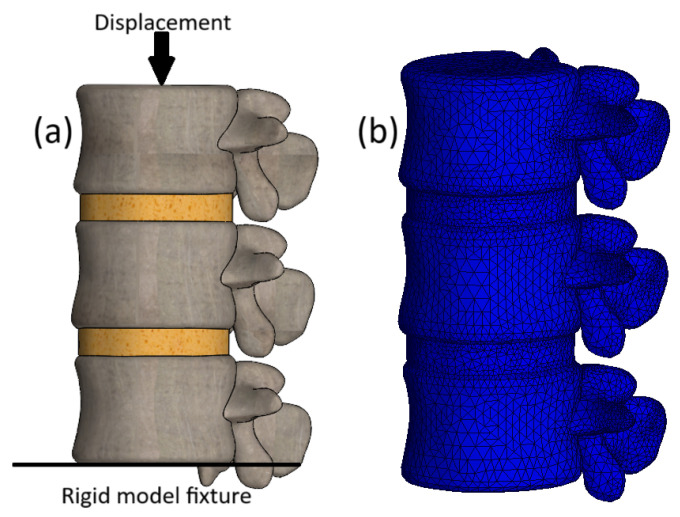
3D model of a healthy lumbar spine: (**a**) Loading scheme; (**b**) Finite element mesh.

The loading configuration is illustrated in Figure 3a. The inferior surface of the L3 vertebra was fully constrained to prevent any motion, while a uniform prescribed displacement of 2.5 mm was applied directly to the superior endplate of L1 across all models developed in this study. The loaded surface was defined as a rigid kinematic boundary (equivalent to a rigid plate), ensuring all nodes moved uniformly. This combination of fixed inferior constraint and rigid displacement loading is known to influence apparent segment stiffness and reaction forces by restricting global deformation modes and enforcing uniform axial compression.

Alternative boundary condition formulations, such as force-controlled loading, compliant endplate constraints, or follower load application, would allow different load-sharing mechanisms and deformation patterns to develop, potentially affecting absolute stiffness and reaction force magnitudes. However, since the present study applies identical boundary conditions across all degeneration stages, the observed comparative trends—driven by progressive disc height reduction and material property changes—remain internally consistent and suitable for relative analysis.

The prescribed 2.5 mm axial displacement was selected to represent a moderate, non-extreme compression range for a two-disc lumbar segment (L1–L3). This corresponds to approximately 1–1.5 mm of disc compression per level [37], which is consistent with expected deformation under moderate compressive loading in neutral posture.

Displacement-controlled loading was used to impose identical kinematic conditions across all models, enabling direct comparison between healthy and degenerated discs and isolating the effects of geometric and material changes on stress distribution. This approach improves reproducibility and numerical stability by avoiding stiffness-dependent deformation variability typical of force-controlled loading. Follower loads and muscle forces were omitted to maintain a controlled setup; although this affects absolute stiffness and load sharing, comparative trends across degeneration stages remain valid.

Facet articulation in the present study was represented using two formulations: (1) no facet contact in the healthy and mild stages; and (2) facet contact in the moderate and severe stages. Under pure axial compression in neutral posture, facet joints are expected to contribute minimally to load sharing in healthy lumbar segments [38]. Thus, the no-contact assumption (the facet contact solver was inactive) provides a reasonable approximation of low facet involvement during early degeneration. Conversely, disc height loss in advanced degeneration can reduce joint space and promote posterior approximation; the contact formulation therefore serves as an upper-bound scenario that intentionally exaggerates posterior load transfer [39] to explore its mechanical consequences. Therefore, the observed load shift to posterior elements in moderate and severe degeneration stages also results from this degeneration-driven modeling approach. The coefficient of friction was set to 0.5 for the moderate degeneration model (corresponding to Grade 2 per Weishaupt classification) and 0.9 for the severe degeneration model (Grade 4, simulates near-complete cartilage loss and high frictional resistance typical of advanced osteoarthritis) [39].

The finite element mesh consisted of quadratic 10-node tetrahedral elements (Figure 3b), chosen to accurately represent complex vertebral geometry, particularly in the posterior elements and endplate interfaces. This formulation enables efficient local refinement, minimizes distortion, and ensures stable and accurate stress computation. Mesh convergence was verified through successive refinements, with changes in maximum von Mises stress and displacement remaining below 5% (Table 3). All models exhibited sufficient spatial resolution, with element sizes ranging from 0.0029 to 3 mm. Although the severe model contains fewer elements due to geometric volume loss, the element size in the L1–L2 disc region was controlled to maintain sufficiently fine local mesh density in the remaining thin disc, and the reported peak AF stress (6.3 MPa) remained within the <5% convergence threshold under mesh refinement.

Each simulation required approximately 345 min of computation time on a workstation equipped with a 3.2 GHz quad-core AMD Ryzen 5 processor and 16 GB of RAM.

### 2.6. Modeling Assumptions and Their Implications for Interpretation

The finite element framework employed in this study relies on a set of deliberate modeling assumptions introduced to enable a controlled and systematic investigation of degeneration-related load redistribution in the lumbar spine. These assumptions ensure numerical robustness, minimize geometric and material uncertainty, and allow consistent comparison across degeneration stages under identical loading conditions. Table 4 summarizes the key simplifications, their rationale, and implications.

Although the assumptions and lack of direct experimental validation prevent the model from being a fully comprehensive physiological representation, it provides a robust numerical basis for identifying characteristic stress-transfer patterns in progressive disc degeneration. Results should be interpreted as relative computational indicators rather than definitive physiological predictions.

Within this framework, the model elucidates how coupled geometric and material changes (disc height reduction, nucleus pulposus volume loss, and tissue stiffening) affect load sharing between the disc, vertebral bodies, and posterior elements. The consistent parametric structure enables transparent examination of these relative influences across degeneration stages.

## 3. Results and Discussion

### 3.1. Stress Analysis Across Degeneration Stages in the L1–L3 Segment

**Figure 4:**
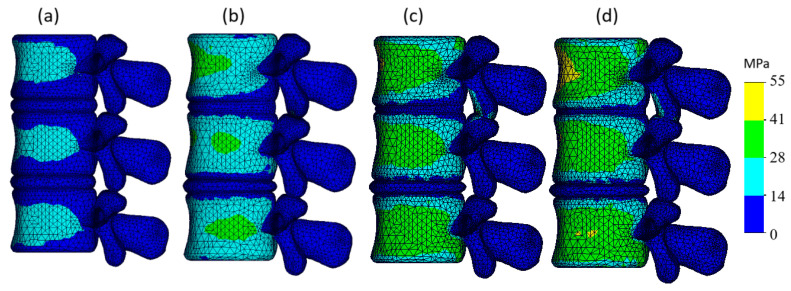
Von Mises stress plots of the three-dimensional model with different stages of IVD degeneration: (**a**) Healthy IVD model; (**b**) Model with mild IVD degeneration; (**c**) Model with moderate IVD degeneration; (**d**) Model with severe IVD degeneration.

The von Mises stress plots for models with different degeneration types in case of 2.5 mm displacement are presented in Figure 4.

In the healthy model (Figure 4a), most stress is concentrated in the cortical shell of the vertebral bodies with stress magnitudes predominantly ranging between 14 and 28 MPa (cyan zones). The distribution appears uniform, while stress within the intervertebral discs and posterior elements remains minimal, typically below 14 MPa (blue zones).

In the mild degeneration model (Figure 4b), stress increases slightly in the vertebral bodies, with more prominent green areas (peak value reaches ~40 MPa), especially in the central areas of the vertebral bodies. With moderate degeneration (Figure 4c), stress further intensifies and green areas (~40 MPa) become more widespread in the cortical regions; however, higher levels (above 41 MPa) on the cortical shell are still not observed. In the severe degeneration model (Figure 4d), the stress becomes even more pronounced: the cortical bone exhibits larger green regions approaching the 41 MPa threshold; however, even in this case, the peak cortical stress does not exceed 55 MPa.

In the healthy and mildly degenerated models, the posterior elements remained largely unloaded, whereas in the moderate and severe stages these structures began to carry noticeable stress, reaching up to 55 MPa in the severe case. This behavior results from the axial displacement-controlled loading used in the present study: under pure compression, the facet joints do not engage in the healthy configuration, and their involvement appears only when disc height loss reduces the joint space. The simplified representation of facet articulation employed here does not reproduce physiological facet mechanics, as no contact formulation was solved and neither contact area nor frictional behavior was modeled. Therefore, the absolute stress magnitudes should be interpreted cautiously. Nevertheless, the increase in posterior loading with progressive disc height reduction reflects a mechanically plausible consequence of reduced joint spacing under degeneration.

### 3.2. Analysis of Reaction Forces Under Different Degrees of Disc Degeneration

**Figure 5:**
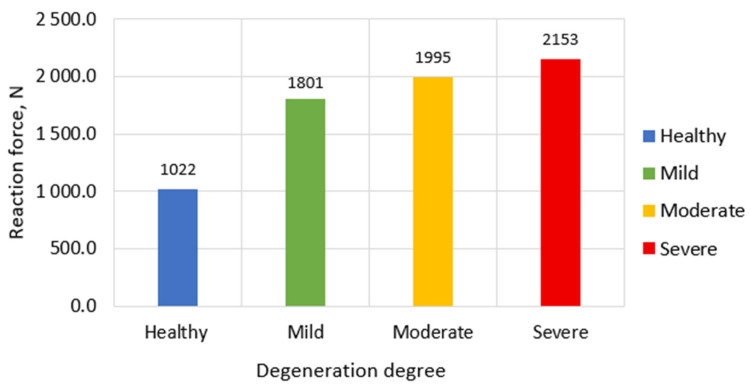
Reaction forces on upper L1 vertebral surface in case of different IVD degeneration degrees.

Figure 5 illustrates the reaction forces measured on the upper surface of the L1 vertebra in response to applied displacement across different stages of intervertebral disc (IVD) degeneration. As observed, the magnitude of reaction force progressively increases with the severity of degeneration.

It should be noted that in this study, we consider the reaction forces as comparative indicators of relative segmental stiffness, rather than as absolute biomechanical measures requiring additional normalization. In the healthy disc model, the total reaction force remains at approximately 1022 N, which aligns with expected biomechanical responses under physiological loading. For mild degeneration, this value increases to around 1801 N, reflecting the initial rise in disc stiffness and reduction in energy absorption capacity. In the case of moderate degeneration, the reaction force further elevates to approximately 1995 N, while in the severe degeneration scenario, the maximum reaction force reaches nearly 2153 N.

The most significant jump in reaction force occurs between the healthy and mildly degenerated models, increasing almost twofold—from 1022 N to 1801 N. This sharp rise is primarily associated with the stiffening of the annulus fibrosus and the reduction in disc height, which together impair the nucleus pulposus’s ability to effectively dissipate compressive loads.

In contrast, the transition from mild to moderate degeneration (1801 N to 1995 N), and further to severe degeneration (2153 N), results in a more gradual increase. This plateauing effect suggests that beyond a certain threshold of structural degradation, additional increases in stiffness do not proportionally raise the reaction force. This phenomenon can be explained by the redistribution of mechanical load: as the disc height—particularly in the L1–L2 region—diminishes, the posterior elements begin to bear a greater share of the compressive load. This compensatory mechanism reduces the rate at which reaction force increases with degeneration, as part of the load is offloaded onto these secondary load-bearing structures.

**Figure 6:**
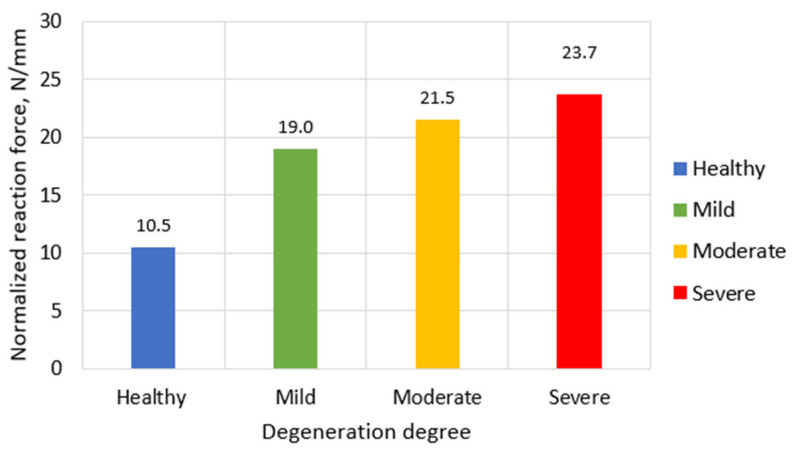
Normalized reaction forces on upper L1 vertebral surface in case of different IVD degeneration degrees.

To account for geometric changes across degeneration stages, reaction forces were normalized by the total vertical height of the L1–L3 segment, measured from the inferior surface of L3 to the superior surface of L1. The normalized values were healthy (97 mm) → 10.5 N/mm; mild degeneration (95 mm) → 19.0 N/mm; moderate degeneration (93 mm) → 21.5 N/mm; severe degeneration (91 mm) → 23.7 N/mm. This normalization reveals that effective segmental stiffness increases by approximately 126% from healthy to severe degeneration, reflecting the combined effects of material stiffening and geometric compaction (Figure 6).

These findings highlight the biomechanical complexity of degeneration progression and emphasize the need to consider the entire spinal segment, rather than the intervertebral disc alone, in finite element analyses.

### 3.3. Stress Patterns in the Intervertebral Disc Across Degeneration Stages

**Figure 7:**
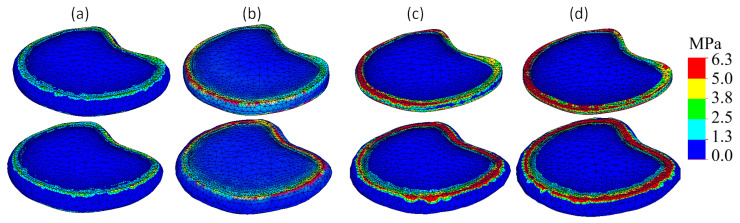
Von Mises stress plots: (**a**) Healthy IVD model; (**b**) Model with mild IVD degeneration; (**c**) Model with moderate IVD degeneration; (**d**) Model with severe IVD degeneration. Upper row presents L1–L2 IVD; bottom row presents L2–L3 IVD.

Figure 7 presents von Mises stress distributions within the L1–L2 and L2–L3 intervertebral discs under 2.5 mm compression for all degeneration stages.

In the healthy model (Figure 7a), stress remains evenly distributed, with peak values localized at the periphery of the annulus fibrosus, not exceeding 1.5 MPa. The nucleus pulposus exhibits minimal stress, consistent with its fluid-like biomechanical behavior and effective load distribution. It should be noted that these simplifications of flat and uniform endplates may introduce localized artifacts in von Mises stress patterns near the bone–disc interface, and these values should therefore be interpreted with caution. However, since the same simplification was applied consistently across all degeneration stages, the comparative trends in stress redistribution remain reliable.

As degeneration progresses, clear shifts in stress distribution patterns are observed. In the mild degeneration model (Figure 7b), stress concentrations begin to form near the posterior-lateral regions of the annulus, indicating altered load transmission pathways. Moderate degeneration (Figure 7c) further intensifies this trend: peak stress zones extend deeper into the annulus and appear bilaterally. In the severe degeneration model (Figure 7d), maximum stress zones become highly localized at the outer edge of the annulus fibrosus, particularly in the posterior region, with von Mises values exceeding 6 MPa.

This shift indicates that progressive degeneration not only reduces disc height but also leads to mechanical overloading of annular fibers, increasing the risk of structural failure, fissures, and herniation.

**Figure 8:**
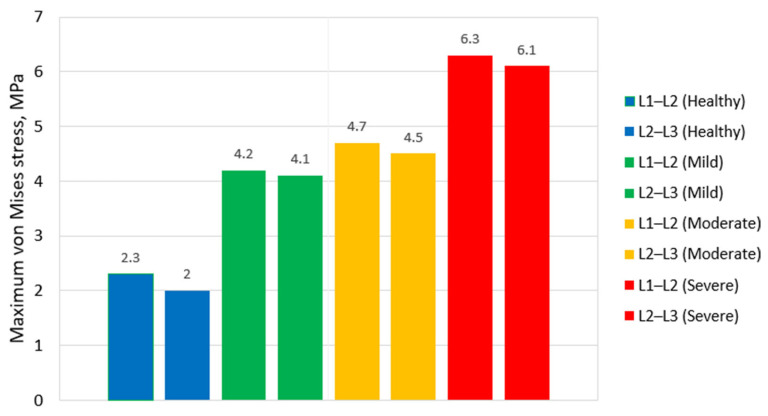
Peak von Mises stress values in the annulus fibrosus under different degeneration scenarios.

The bar chart in Figure 8 quantifies peak von Mises stress values within the annulus fibrosus for all degeneration stages. The stress rises from approximately 2.3 MPa in the healthy disc to 4.2 MPa in mild degeneration, followed by 4.7 MPa in moderate degeneration, and peaking at nearly 6.3 MPa in severe degeneration.

This progressive increase reflects both the compromised energy dissipation ability of the disc and the redistribution of loads from the central nucleus pulposus to peripheral annular structures. The elevated stress values in advanced stages also correlate with increased risk of annular tears and further degeneration of the disc [10].

It is observed that, regardless of degeneration severity, the difference in stress levels between the two discs within the same model remains relatively small. However, the L1–L2 disc consistently exhibits slightly higher maximum stress values compared to L2–L3. This trend is most prominent in the healthy degeneration scenario, where peak stress in L1–L2 reaches 2.3 MPa, while in L2–L3 it is 2 MPa.

The consistently higher stress in L1–L2 is primarily due to the proximity of the applied load, as explained by Saint-Venant’s principle. This proximity effect may be amplified by the displacement-controlled setup with a rigid kinematic boundary (uniform endplate motion) and would likely be reduced under force-controlled or follower-load conditions that allow more natural coupled motions and load sharing. Because the compressive displacement is applied to the superior surface of L1, the adjacent L1–L2 disc undergoes greater localized deformation, while the L2–L3 disc is partially shielded by load dissipation through the upper vertebra. This behavior highlights the influence of boundary conditions in finite element analyses.

**Figure 9:**
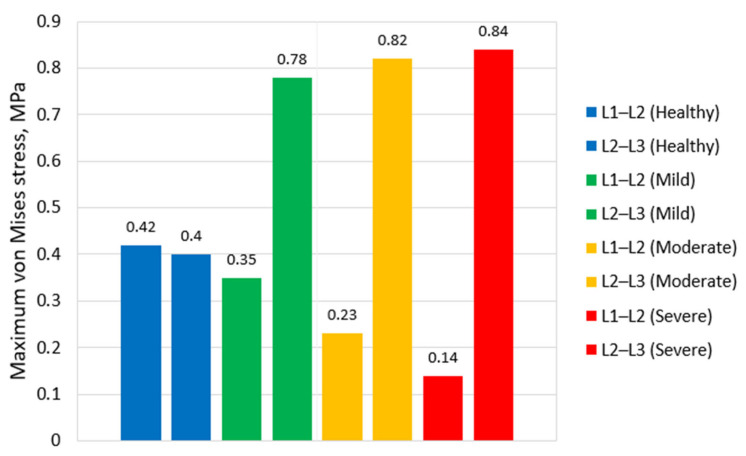
Peak von Mises stress values in the nucleus pulposus under different degeneration scenarios.

Figure 9 illustrates the maximum von Mises stress values in the nucleus pulposus of the L1–L2 and L2–L3 discs for different stages of IVD degeneration. In the healthy models, the peak stresses in the nucleus pulposus of both discs are nearly identical (0.42 MPa for L1–L2 and 0.40 MPa for L2–L3). In mild degeneration at L1–L2, the stress in the degenerated L1–L2 nucleus slightly decreases to 0.35 MPa, consistent with early loss of hydrostatic capacity. Interestingly, the adjacent intact L2–L3 disc experiences a marked increase in nucleus stress (up to 0.78 MPa), likely due to increased load transmission through the mildly stiffened L1–L2 segment under the fixed displacement boundary condition. This effect diminishes in moderate and severe degeneration as the L1–L2 disc loses most of its load-bearing capacity, shifting stresses primarily to the annulus and posterior elements.

However, in the moderate and severe degeneration stages, a sharp decline is observed—stress drops to just 0.23 MPa for L1–L2 (moderate degeneration) and 0.14 MPa for L1–L2 in case of severe degeneration degree. This significant reduction is associated with the collapse of the nucleus pulposus structure and its replacement by fibrotic or calcified tissue. As the nucleus loses its hydrostatic pressure-retaining properties, it no longer effectively transmits compressive loads, resulting in a stress shift toward the annulus fibrosus and posterior elements. Thus, the stress dynamics in the nucleus pulposus across degeneration stages reflect the progressive loss of its biomechanical function, transitioning from a primary load-bearing component to a largely inactive remnant in the degenerated disc.

### 3.4. Results Verification and Discussion

Direct quantitative validation of the present FE model is constrained by the absence of reference data that reproduce the same geometry, degeneration pattern, and boundary conditions used in this study. Existing investigations—whether computational or experimental—differ substantially in anatomical detail, modelling assumptions, loading strategies, and degeneration definitions, which prevents a strict one-to-one comparison with the current subject-specific two-level model. In such situations, verification is appropriately based on demonstrating that the predicted stresses fall within biomechanically reasonable ranges and that the overall mechanical trends align with established understanding of lumbar load transfer, rather than on exact numerical matching with any single dataset.

While direct experimental deformation–load validation was not possible, the model was validated through multi-metric consistency with published FE and experimental trends.

The reaction forces obtained for the healthy disc model (~1022 N at 2.5 mm displacement) closely match experimental lumbar stiffness measurements reported by Park et al. [29]. Similarly, stress distributions observed in the cortical shell and annulus fibrosus are consistent with the finite element studies by [27,28]. Cortical shell stresses (14–55 MPa) are consistent with previously published ranges (8–61 MPa [18] and 7.4–64 MPa [40]), indicating that the model does not produce unrealistically high stress concentrations under axial compression.

For healthy discs, the model’s IDP estimates (approximately 0.5–1.0 MPa under displacement-controlled compression) align closely with in vivo measurements reported by [41], where standing postures yielded 0.5 MPa at L4–L5. Similarly, [42] documented baseline IDP values of 0.3–0.5 MPa in healthy individuals during standing, supporting the model’s representation of uniform load distribution in non-degenerated states. In degenerated scenarios, the observed 70% reduction in nucleus pulposus stress across mild to severe stages corresponds to IDP declines of 40–70% in cadaveric studies by [43], where Pfirrmann-graded discs showed IDP drops from 0.4–1.3 MPa (healthy) to 0.1–0.8 MPa (degenerated), alongside height losses of 0.2–1.0 mm. This is further corroborated by in vivo data from [42], indicating 40–60% IDP reductions (to 0.1–0.3 MPa) in degenerative disc disease patients with MRI grading. The model’s 160% increase in annulus fibrosus stress mirrors elevated stresses (3–10 MPa) and failure loads in degenerated annuli [44], with stiffness increases of 10–20%. Additionally, trends in range of motion (ROM)—initial increases followed by decreases—align with [45], reporting 20–30% ROM elevations at moderate degeneration (grade 4) and 15–25% reductions at severe stages (grade 5). Posterior load shifts in advanced degeneration are consistent with [46], where telemetry data showed IDP in sitting (0.2–0.6 MPa) and standing (0.3–0.4 MPa) for degenerative disc diseases, indicating no exacerbated risk from sitting compared to standing. A further decline in nucleus pressure has been reported during early degeneration, corresponding to 25–30% reductions in IDP from 0.25–0.45 MPa to 0.19–0.34 MPa [47].

Overall, these comparisons affirm the model’s qualitative and quantitative trends in load redistribution and place the predicted responses within biomechanically plausible ranges reported in vivo and in cadaveric studies. Absolute magnitudes should still be interpreted cautiously, as simplifications such as isotropy and idealized boundary conditions can affect peak values, as discussed in the limitations.

The variation of peak stress values in the nucleus pulposus requires separate consideration and detailed explanation. According to different sources, disc degeneration may lead either to an increase [27] or a decrease [10] in intradiscal stresses, while some studies report opposite trends. In our work, both patterns were observed, and each requires careful interpretation. In the healthy model, the peak stresses in both discs are nearly identical (0.42 and 0.40 MPa). Under mild degeneration, however, the stress in L2–L3 nearly doubles, whereas the stress in L1–L2 slightly decreases, which aligns with the trends described in [10]. As degeneration progresses, stresses in the deteriorated disc continue to decrease, while the intact L2–L3 disc exhibits only a minor increase.

The non-monotonic changes in NP stress across degeneration stages can be explained by the shift in its mechanical role. In the healthy state, the NP behaves as a hydrated, hydrostatic structure that carries a large portion of the axial load, which leads to relatively high internal stress. During mild degeneration, the NP still retains its ability to sustain pressure, while the reduction in disc height and the early stiffening of the annulus can increase nucleus compression, resulting in a modest stress elevation at the adjacent level.

With progression to moderate and severe degeneration, the NP loses volume and hydration and becomes mechanically less distinct from the surrounding annulus. As a result, it can no longer sustain hydrostatic pressure and contributes less to load bearing, so NP stress decreases despite the overall increase in segmental stiffness. The load path shifts toward the stiffer annulus fibrosus and endplates, leading to a more deviatoric NP response. These findings underscore the value of parametric studies, since stress responses depend on the coupled effects of tissue properties and geometry, which can generate markedly different mechanical patterns.

### 3.5. Sensitivity Analysis

To assess the robustness of the model and the influence of input parameter variations on the observed trends, a limited sensitivity analysis was performed. Key parameters were varied around their baseline values, and the impact on peak von Mises stresses in the annulus fibrosus (AF) and nucleus pulposus (NP), as well as on the total reaction force at the superior surface of L1, was evaluated. The results are presented in Table 5.

The following parameters were tested in the healthy and severe degeneration configurations:

- Disc height: ±10% from baseline (healthy: 10 mm → 9 mm/11 mm; severe: 4 mm → 3.6 mm/4.4 mm).
- Nucleus pulposus volume (cross-sectional area): ±15% from baseline.
- Annulus fibrosus stiffness (Mooney–Rivlin constants *C*_1_ and *C*_2_): ±20% from baseline values.

All other parameters (boundary conditions, mesh setup, material properties of bone/endplates) remained unchanged. Simulations were performed using the same displacement-controlled loading (2.5 mm axial compression) as in the main study.

The conducted sensitivity analysis helped identify the following key observations:

- The model is most sensitive to disc height variations: a 10% reduction increases peak AF stress by 13–18% and total reaction force by 15–23%, while a 10% increase reduces these values accordingly. This confirms disc height as one of the dominant geometric factors influencing segmental stiffness and annulus loading.
- Nucleus pulposus volume has a moderate effect, primarily on NP stress (changes up to ±13–15%), with minimal impact on AF stress (±3–4%) and reaction force (±8–11%).
- Annulus fibrosus stiffness (*C*_1_ and *C*_2_) exerts the strongest influence on peak AF stress (up to ±26%) and reaction force (±13–19%), but has negligible effect on NP stress (±2–6%).
- Importantly, all qualitative trends remain consistent across the tested ranges: progressive degeneration still leads to increased AF stress, decreased NP stress, and a shift of load toward posterior elements. No reversal of trends was observed.

Stress evaluation in the present study is primarily based on peak von Mises values. This choice was motivated by the highly localized nature of stress concentrations observed in degenerated discs, particularly within the annulus fibrosus following nucleus pulposus volumetric collapse. Such local stress maxima are mechanically relevant, as they are more closely associated with the initiation of annular tears and structural failure than spatially averaged measures.

Despite the deliberate modeling simplifications adopted in this study, several key findings can be considered robust. In particular, the qualitative trends of load redistribution with progressive disc degeneration—namely the increase in annulus fibrosus stress, the marked reduction of nucleus pulposus stress, and the posterior shift of load sharing in advanced stages—remain consistent across all tested parameter variations and degeneration grades. Sensitivity analysis confirmed that these trends persist under ±10–20% variations in disc height, nucleus volume, and annulus stiffness, indicating that the observed mechanisms are primarily driven by geometric collapse and relative tissue stiffening rather than by secondary stabilizing structures.

At the same time, results related to absolute segment stiffness, reaction force magnitude, and the quantitative contribution of posterior elements are likely sensitive to the omission of ligaments and follower load. Ligamentous tension and muscle-induced follower loads are known to influence spinal stability and load-sharing, particularly under physiological posture and motion. Their exclusion may therefore affect the absolute values of reaction forces and posterior element stresses. However, because these simplifications were applied consistently across all degeneration stages, the comparative interpretation of degeneration-driven mechanical trends remains valid.

## 4. Conclusions

This study presents a robust parametric finite element framework for evaluating load redistribution in progressive lumbar disc degeneration, demonstrating significant shifts in biomechanical behavior across healthy, mild, moderate, and severe stages. Key findings include a 175% increase in annulus fibrosus von Mises stresses, elevating risks of tears and herniation; a ~70% decrease in nucleus pulposus stresses, indicating loss of hydrostatic function; and a progressive load transfer to posterior elements, suggesting mechanically unfavorable load redistribution patterns that may be associated with conditions such as facet joint degeneration or vertebral bone deconditioning. The non-monotonic nucleus stress patterns—initial rise in adjacent intact discs due to early stiffening, followed by sharp declines—highlight the complex interactions of geometric and material changes. The observed reduction in nucleus pulposus stress in advanced degeneration should not be interpreted as stress relief. Instead, it reflects a loss of the nucleus load-bearing function due to volumetric collapse, whereby the nucleus becomes mechanically embedded within the annulus fibrosus and no longer participates effectively in hydrostatic load sharing.

Verified against in vivo intradiscal pressure reductions (40–70%) and cadaveric data, and confirmed robust through sensitivity analysis (±10–20% parameter variations yielding <26% quantitative changes), these trends provide reliable insights into degeneration mechanics. Unlike prior single-factor models, presented approach offers a comprehensive, reproducible pipeline that elucidates coupled mechanisms and adjacent-segment effects, empowering clinicians to better assess progression risks and tailor interventions, while guiding biomedical engineers in designing more effective implants and therapies.

While simplifications (e.g., isotropic materials, no poroelasticity) limit absolute physiological accuracy, their consistent application ensures valid comparative analysis. These interpretations should be understood within the scope of the present simplified model, which does not include ligamentous structures, active muscle forces, or population-level anatomical variability, and therefore aims to describe relative mechanical trends rather than direct clinical outcomes. Future extensions could incorporate anisotropic fiber models, follower loads, or multi-subject geometries to further enhance predictive power and clinical applicability.

## Figures and Tables

**Table 1:** Mechanical properties of bone and endplates.

Model Component	Young’s Modulus, MPa	Poisson’s Ratio	Yield Strength, MPa
Cortical bone	8000	0.3	64
Cancellous Bone	100	0.3	
Posterior elements	3500	0.3	
Endplates	24	0.4	

**Table 2:** Elastic constants of IVD components accordingly to degeneration [19,26,27,28,29].

Degeneration Stage	*C*_1_, MPa (AF)	*C*_2_, MPa (AF)	*C*_1_, MPa (NP)	*C*_2_, MPa (NP)
Healthy	0.18	0.045	0.12	0.030
Mild	0.40	0.100	0.14	0.035
Moderate	0.60	0.150	0.17	0.041
Severe	0.90	0.230	0.19	0.045

**Table 3:** Finite element mesh parameters for calculation cases reflecting different stages of IVD degeneration.

Model	Number of Elements	Number of Nodes	Number of Degrees of Freedom	Element Size, mm	Skewness; Aspect Ratio	Number of Jacobian Points
Healthy	576,345	704,576	2,111,946	0.0029–3	<0.8; <3	16
Mild	559,054	683,457	2,009,363			
Moderate	536,692	656,108	1,922,396			
Severe	509,857	623,296	1,832,490			

**Table 4:** Summary of key modeling simplifications, their rationale, and implications for interpretation.

Simplification	Justification	Implications for Interpretation
Absence of follower load and muscle forces	Avoids additional assumptions related to curvature-dependent load paths and subject-specific muscle activation, preserving a controlled parametric framework.	Load transmission occurs through simplified axial compression; deformation modes and reaction forces may differ from physiological loading scenarios, while comparative degeneration-driven trends remain interpretable.
No ligament structures	Avoids adding nonlinear, tension-only ligaments with subject-specific properties and attachment uncertainty, ensuring a controlled, reproducible comparison across degeneration stages under compression load.	The segmental mechanical response under compression primarily reflects disc- and bone-driven behavior. Ligament-mediated stabilization is not included, so results characterize internal load redistribution in the absence of soft-tissue tension contributions.
Flat endplates	Controls geometric variability and isolates the mechanical contribution of disc degeneration without additional curvature-related effects.	Flattened boundaries may over constrain the segment, restricting small, coupled motions under compression and thereby increasing apparent stiffness and potentially inflating absolute reaction forces.
Homogeneous cancellous bone representation	CT-based heterogeneity was not the focus; uniform cancellous properties improve numerical robustness and reduce computational cost.	Local stress heterogeneity within vertebral bodies is not resolved; the vertebral body response represents an effective, averaged mechanical behavior.
Isotropic hyperelastic annulus fibrosus	Prioritizes numerical stability and enables a consistent parametric degeneration framework; explicit fiber-level anisotropy was beyond the intended scope.	The annulus response represents bulk mechanical behavior; direction-dependent fiber effects are not explicitly captured, which may influence absolute stress magnitudes.
Fixed inferior constraint and rigid displacement loading	Ensures numerical stability and reproducible kinematic conditions across all degeneration stages, allowing controlled comparison of disc-driven mechanical effects.	The flattened external boundaries may increase apparent stiffness. However, relative mechanical trends across degeneration stages remain valid due to consistent application.
Single-sample geometry	Reduces inter-subject anatomical variability and isolates the mechanical effects of progressive disc degeneration, enabling a controlled comparison across degeneration stages without confounding geometric differences.	The results reflect degeneration-driven mechanical trends within a fixed anatomical configuration and may not capture population-level variability associated with subject-specific geometry; therefore, findings should be interpreted as mechanistic rather than statistical generalizations.

**Table 5:** Results of the sensitivity analysis (percentage change relative to baseline values).

Parameter Varied	Variation	Configuration	% Change in Peak AF Stress (L1–L2)	% Change in Peak NP Stress (L1–L2)	% Change in Total Reaction Force
Disc height	+10%	Healthy	−14%	+9%	−16%
Disc height	–10%		+18%	−11%	+23%
Disc height	+10%	Severe	−13%	+6%	−12%
Disc height	–10%		+16%	−8%	+15%
Nucleus pulposus volume	+15%	Healthy	−3%	+13%	−8%
Nucleus pulposus volume	–15%		+4%	−14%	+11%
AF stiffness (*C*_1_ and *C*_2_)	+20%	Healthy	+21%	−6%	+15%
AF stiffness (*C*_1_ and *C*_2_)	–20%		−17%	+5%	−13%
AF stiffness (*C*_1_ and *C*_2_)	+20%	Severe	+26%	−4%	+19%
AF stiffness (*C*_1_ and *C*_2_)	–20%		−20%	+2%	−16%

## Data Availability

Data presented in this study are available on request from the corresponding author.
